# Distribution pattern of soil nematode communities along an elevational gradient in arid and semi-arid mountains of Northwest China

**DOI:** 10.3389/fpls.2024.1466079

**Published:** 2024-10-14

**Authors:** Jingliang Chen, Yafeng Zhang, Chao Liu, Lei Huang

**Affiliations:** ^1^ Key Laboratory of Ecological Safety and Sustainable Development in Arid Lands/Shapotou Desert Research and Experiment Station, Northwest Institute of Eco-Environment and Resources, Chinese Academy of Sciences, Lanzhou, China; ^2^ University of Chinese Academy of Sciences, Beijing, China; ^3^ Ningxia Luoshan National Nature Reserve Management Bureau, Wuzhong, China; ^4^ College of Forestry and Prataculture, Ningxia University, Yinchuan, China

**Keywords:** soil nematodes, diversity, richness, maturity indices, soil factors

## Abstract

Soil nematodes are the most abundant soil metazoans, occupying multiple trophic levels in the soil food web and playing an important role in soil function. Research on the biogeographic distribution patterns of soil nematode communities and their drivers has received greater attention. However, the distribution characteristics of soil nematode communities along the elevational gradient in the arid and semi-arid regions of Northwest China remain unclear. In this study, four elevational gradients (1750–1900, 1900–2100, 2100–2350 and 2350–2560 m) were established on Luoshan Mountain, Ningxia, an arid and semi-arid region in Northwest China, and soil nematodes in the soil layers of 0–10, 10–20 and 20–40 cm were investigated using the improved Baermann funnel method. The results revealed a monotonically decreasing trend in the total number of soil nematodes along the elevational gradient and soil layer depth, decreasing by 63.32% to 79.94% and 73.59% to 86.90%, respectively, while the interactions were not obvious. A total of 1487 soil nematodes belonging to 27 families and 32 genera were identified across the elevational gradient, with *Helicotylenchus* as the dominant genus, accounting for 10.43% of the total number of nematodes, and bacterivore nematodes as the main trophic groups, accounting for 32.39% to 52.55% of the relative abundance at each elevation, which increased with increasing elevation. Soil nematode community diversity, richness and maturity indices were relatively low at high elevation and decreased by 44.62%, 48% and 54.74%, respectively, with increasing soil layer depth at high elevations. Compared to low elevations, high-elevation soils experienced greater disturbance, reduced structural complexity and nutrient enrichment of the soil food web, and a shift in soil organic matter decomposition from bacterial to fungal pathways as elevation increased. Finally, redundancy analysis showed that soil pH, bulk density, soil moisture, soil organic carbon, available nitrogen, available phosphorus and available potassium were the main soil factors affecting the composition of soil nematode communities, which well explained the differences in nematode communities at different elevations and soil depths. This study can be used as basic information for further research on soil biota in this mountainous region, expanding our further understanding of the spatial ecology of soil nematodes in the arid and semi-arid mountain ecosystems.

## 1 Introduction

Soil nematodes are a key biome in soil ecosystems and are numerous and diverse, accounting for four-fifths of all animals on land (Li G. X., et al., 2023; Kouser et al., 2021; van den Hoogen et al., 2019). As the most numerous soil metazoans, they exhibit high species diversity, dietary diversity, life history strategy diversity, and functional group diversity (Zhao et al., 2019; Pen-Mouratov et al., 2010). They can range from “colonizers” (i.e., r-strategist) to “persisters” (i.e., K-strategist) in nematode life-history strategies, encompassing all intermediate stages between these two extremes (i.e., “cp “) (Choudhary et al., 2023). Soil nematodes occupy various trophic levels (bacterivores, fungivores, omnivores-predators, and plant parasites (Ma et al., 2022; Yeates et al., 1993) and play multiple roles in the soil food web (nutrient cycling, organic matter decomposition, plant growth regulation and serving as bioindicators of soil health and environmental changes) (Bardgett and van der Putten, 2014; Bongers and Ferris, 1999). They also play crucial roles in improving soil physical structure, promoting nutrient cycling and organic carbon stabilization, and enhancing crop health, thus contributing significantly to soil functions (Bardgett and van der Putten, 2014; Fu et al., 2022; Tu et al., 2022). Soil nematodes also serve as bioindicators of soil health and environmental changes (Li G. X. et al., 2023), and the diversity and community structure can reflect subtle changes in the soil environment, making them important for ecosystem monitoring and management (Shen et al., 2023; Zhang et al., 2021; Inácio et al., 2023). Elevation has been reported to be a significant factor affecting the diversity and abundance of nematode communities because of differing climatic conditions (such as temperature and precipitation) and vegetation types at different elevations (Afzal et al., 2021). However, very few studies have been done on the distribution patterns and influencing factors of soil nematode communities along elevation gradients in mountain ecosystems of arid and semi-arid regions, limiting our understanding of soil biodiversity and ecological processes in these fragile ecosystems (van den Hoogen et al., 2019).

Recent studies on nematode communities across elevation gradients have yielded varied conclusions regarding their distribution patterns and influencing factors. For instance, Zhang et al. (2021) observed a monotonic decrease in nematode diversity along the elevation gradient in Oakley, Great Khingan, China. In contrast, Shen et al. (2023) found a non-monotonic decline in nematode α-diversity, influenced by soil bulk density, moisture, dissolved organic carbon, temperature, pH, nitrate nitrogen, and total phosphorus. Choudhary et al. (2023) and Kashyap et al. (2022) reported a negative correlation between nematode diversity and abundance with elevation in the Himalayas, notably in the Pir-Panjal range (Afzal et al., 2021). In the Andes (southern Ecuador), nematode diversity was unaffected by elevation or soil depth, but community composition varied with elevation, influenced by litter C/N and fungal biomass (Traunspurger et al., 2017). Taylan et al., (2021) identified a ‘hump-shaped’ distribution of nematode species richness at mid-elevations on Mount Ararat, Turkey, while Kergunteuil et al. (2016) reported increased nematode diversity with elevation in the Alps. These conflicting results highlight the need for further investigation into nematode distribution patterns along elevation gradients. Furthermore, most existing studies are concentrated in tropical or temperate regions (Marian et al., 2018; Wu et al., 2022), with relatively few focusing on nematode communities in arid and semi-arid mountain regions. This is especially true for the northwestern arid and semi-arid mountains, where the response of soil nematode communities to elevation changes and the driving factors of these changes requires further investigation. Therefore, more research is needed in mountainous ecosystems to establish general patterns and the influencing factors of nematode community distribution across different elevation gradients (Dong et al., 2017).

Ningxia Luoshan, located in the arid and semi-arid region between the western Ordos Plateau and the northern Yellow River Plateau, serves as an important green ecological barrier and water conservation area in central Ningxia’s arid zone. It plays a critical role in regional biodiversity conservation, preventing soil erosion, and maintaining ecological security. Known as the “Emerald of the Desert” and the “Pearl of the Gobi,” it represents a typical fragile ecosystem of the northwestern arid and semi-arid mountainous region (Chen et al., 2023). At the same time, the vegetation shows distinct vertical distribution: at low elevations, it is primarily perennial herbaceous steppe; at mid-elevations, it consists mainly of evergreen coniferous and broadleaf shrubs; and at high elevations, it features mixed forests dominated by *Picea crassifolia*, *Pinus tabuliformis* and *Populus davidiana*. This area provides a good experimental platform for our research. Therefore, in order to better understand soil biodiversity and ecological processes in the fragile ecosystems of northwestern arid and semi-arid mountains, this study investigates the distribution patterns and influencing factors of soil nematode communities along elevation gradients in Luoshan, Ningxia.

The goals of this study are to provide new insights into the distribution patterns of soil nematodes along elevation gradients in the northwestern arid and semi-arid mountainous ecosystem and to offer scientific support for ecosystem management under global change. The specific objectives of the study are: (1) to determine the distribution patterns of soil nematode communities along the elevation gradient in the typical arid mountainous ecosystem; (2) to analyze whether elevation, soil depth, and the interaction of elevation and soil depth have an effect on soil nematode community diversity, richness, maturity, and total number of nematodes; (3) to assess the stability of the soil ecosystem along the elevation gradient using nematode faunal analysis; and (4) to identify the relationship between soil nematode communities and soil physicochemical properties and determine the main soil factors influencing nematode community distribution.

## 2 Materials and methods

### 2.1 Study site

**Figure 1:**
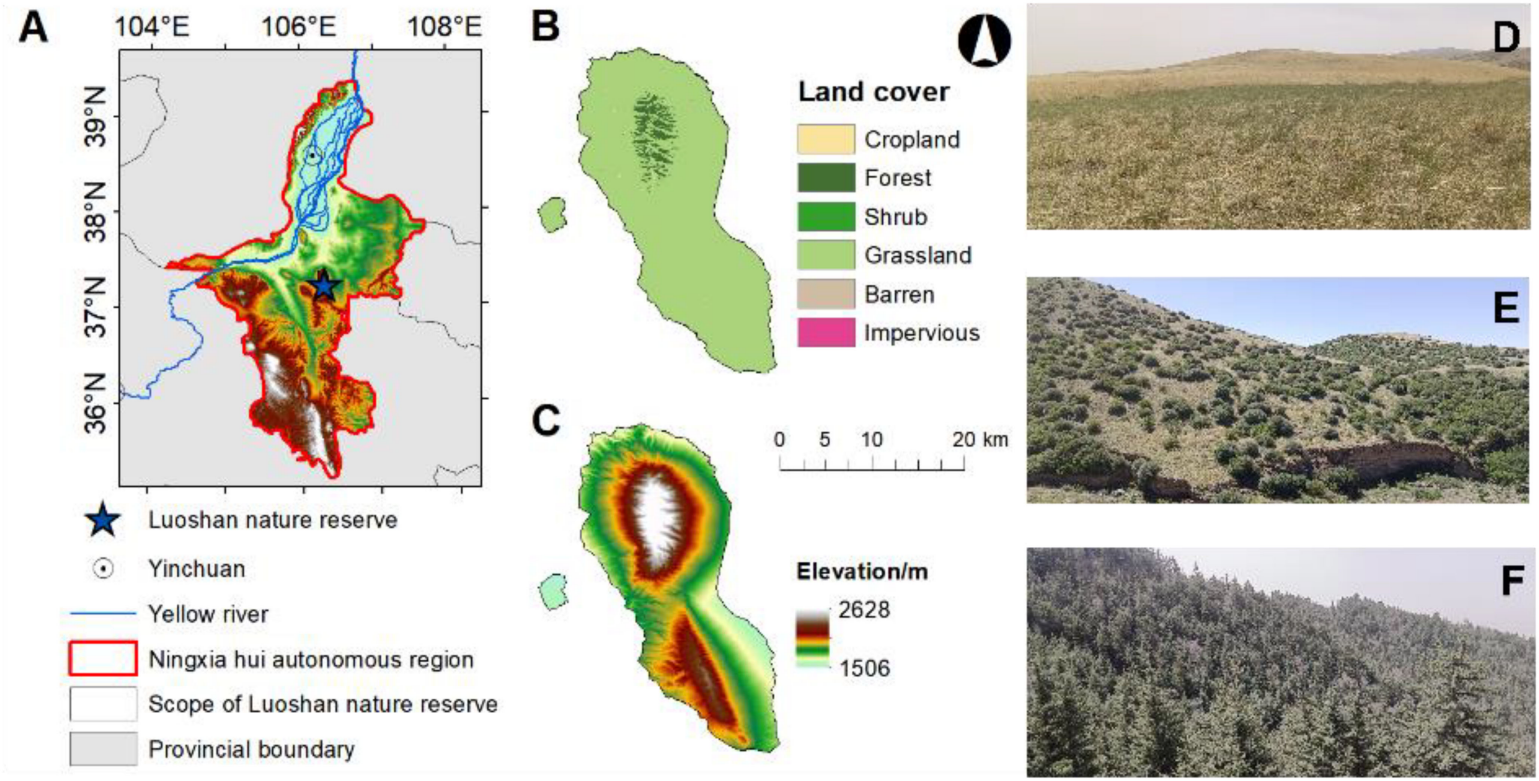
Location of Luoshan in Ningxia Hui Autonomous Region **(A)**; Vertical distribution of vegetation in Luoshan, Ningxia **(B)**; Elevation diagram of Luoshan, Ningxia **(C)**; Schematic vegetation along the elevation gradient at Luoshan, Ningxia **(D)**; In elevation 1750-1900 m,it is mainly Perennial herbaceous steppe **(D)**; In 1900-2100 m, it is mainly evergreen coniferous and broad-leaved scrub dominated by *Ostryopsis davidiana* and *Cotoneaster multiflorus*, etc. **(E)**; In the 2100-2560 m, the mixed coniferous and broad-leaved forests were mainly dominated by *Picea crassifolia*, *Pinus tabuliformis* and *Populus davidiana* **(F)**.

The study area was located in the Luoshan National Nature Reserve (37°11’-37°25’N, 106°04’-106°24’E) of Ningxia Hui Autonomous Regions of China (**Figure 1A**). The reserve with a length of 36 km from north to south, a width of 18 km from east to west and an elevation of 1560-2624.5 m above sea level, and a total area of 33,710 hm^2^ (**Figure 1C**). This area belongs to the arid and semi-arid zone in the western part of the Ordos Plateau and the northern part of the Yellow River Plateau. It has a mesothermal arid continental climate, characterized by an annual average of 2,881.5h of sunshine, large annual and diurnal variations in temperature, and low and concentrated precipitation with an annual mean of 262.5 mm. The soil is mainly dominated by gray-brown soil and gray-calcium soil. The vertical distribution of vegetation is distinct (**Figure 1B**): at an elevation of 1750-1900 m, the area is mainly covered by perennial herbaceous steppe (**Figure 1D**); Between 1900-2100 m, the vegetation is mainly dominated by evergreen coniferous and broadleaf scrub such as *Ostryopsis davidiana* and *Cotoneaster multiflorus* (**Figure 1E**); At elevations ranging from 2100-2560 m, mixed coniferous and broadleaf forests prevail, dominated by *Picea crassifolia*, *Pinus tabuliformis* and *Populus davidiana* (**Figure 1F**; **Supplementary Table S1**).

### 2.2 Soil sampling

In July 2022, we established four elevation gradients (E1: 1750-1900 m, E2: 1900-2100 m, E3: 2100-2350 m, E4: 2350-2560 m) in the Luoshan National Nature Reserve, Ningxia, where terrain factors such as slope shape, slope gradient, and slope aspect were consistent. At each elevation gradient, we randomly selected three replicate plots for soil sampling (1m × 1m for E1, 5 m × 5 m for E2, and 20 m × 20 m for E3 and E4). We randomly selected three replicate plots for each elevation gradient (E1 plots were 1m×1m, E2 plots were 5m×5m, and E3 and E4 plots were 20m×20m) and collected soil samples from three random quadrats within each plot. After removing the litter and humus layers from each quadrat, we collected soil samples at depths of 0-10 cm, 10-20 cm, and 20-40 cm, taking five samples from each layer. In total, 540 soil samples were collected (4 elevation gradients × 3 replicate plots × 3 quadrats × 3 depths × 5 samples per depth), and 15 soil samples from each layer at each plot were mixed to form a composite sample, resulting in 36 composite samples (4 elevation gradients × 3 replicate plots × 3 composite samples). Gravel, roots, and animal residues were removed. The composite soil samples were divided into three portions. One portion was stored at 4°C for the determination of available nitrogen (AN), available potassium (AK), and available phosphorus (AP). Another portion was taken back to the laboratory, air-dried, ground, and passed through a 2 mm sieve for the determination of soil organic carbon (SOC), total nitrogen (TN), total phosphorus (TP), and total carbon (TC). The remaining portion was kept at 4°C for the measurement and analysis of soil nematodes.

### 2.3 Soil physicochemical properties

We used the cutting ring method to measure soil bulk density (BD) and the gravimetric method to determine soil moisture (SM). A pH meter (AS 600, Shanghai) was inserted into a soil-water suspension with a ratio of 1:5 to measure pH. SOC and TC were measured using the K_2_Cr_2_O_7_ oxidation-external heating method. TN was determined using the H_2_SO_4_ digestion-Kjeldahl method (Kjeldahl, 1883; Choudhary et al., 2023). TP was measured using the alkaline NaOH fusion- molybdenum-antimony anti-spectrophotometry. AN was determined using the alkaline hydrolysis diffusion method. AK was extracted with CH_3_COONH_4_ solution and measured using the flame photometry method. AP was extracted with NaHCO_3_/NaF hydrochloric acid and measured using the molybdenum-antimony anti-colorimetry method (Li et al., 2007).

### 2.4 Soil nematode extraction and identification

In the laboratory, we separated soil nematodes using a modified Baermann funnel method (Zhang et al., 2023). The isolated soil nematodes were identified, classified, and counted using a stereo microscope or an inverted microscope (Nikon Ti2-U, 400× and 1000× magnification). The references used for identification included “Pictorial Keys to Soil Animals of China” (Yin, 2000), and the works of Ahmad and Jairajpuri (2010), as well as the Nemaplex database (http://nemaplex.ucdavis.edu/). Nematodes were classified to the genus level (Jairajpuri and Ahmad, 1992). For each sample, we randomly selected 100 nematodes for identification; if the sample contained fewer than 100 nematodes, all nematodes were identified (Huo et al., 2021). Soil moisture was measured using the drying method, and nematode density was converted to individuals per 100 g of dry soil. Based on feeding habits and morphological characteristics such as the head and oral cavity, nematodes were categorized into four trophic groups: bacterivores, fungivores, omnivores-predators, and plant parasites (Yeates et al., 1993).

Each nematode has a different life history c-p value. Nematodes with c-p values of cp1 to cp2 are r-strategists, characterized by short life cycles, tolerance to disturbances, and an ability to survive in harsh environments. In contrast, nematodes with c-p values of cp3 to cp5 are k-strategists, which have long life cycles, reduced reproductive rates, sensitivity to disturbances, and a tendency to thrive in stable, undisturbed systems (Bongers and Bongers, 1998; Franco et al., 2021).

### 2.5 Nematode community analysis

The classification of nematode dominance was based on the following criteria: taxa with individuals comprising more than 10% of the total number were considered dominant (+++); those comprising 1% to 10% were considered common (++); and those with individuals making up less than 1% were considered rare (+).

The analysis of soil nematode community structure used the nematode species diversity indices and the functional indices of nematode community structure. The diversity characteristics of soil nematode communities were commonly represented by indices like Shannon-Wiener diversity index (H’), Pielou’s evenness index (J’), Simpson dominance index (λ) and Margalef richness index (SR). Indices like Maturity index (MI), Plant parasite index (PPI), Wasilewska index (WI), Nematode channel ratio (NCR), Enrichment index (EI) and Structure index (SI) were often used to represent the functional structure characteristics of soil nematode communities. The formulas were as follows:

1. Shannon-Wiener diversity index:


H'=−∑​pi×ln(pi) 

     (Shannon and Weaver, 1949)

2. Pielou’s evenness index:


$$J'=\frac{H}{\text{ln}\left(S\right)}$$

        (Pielou, 1966)

3. Simpson dominance index:


$$\lambda =\sum {\left(pi\right)}^{2}$$

        (Simpson, 1949)

4. Margalef richness index:


$$SR=\frac{S-1}{lnN}$$

        (Margalef, 1958)

where *Pi=ni/N*, *ni* is the number of individuals in taxon *i*, *N* is the total number of individuals in all taxa of the community, and *S* is the number of taxa.

5. Maturity index:


MI=∑​ci×pi

(Bongers, 1990)

where *ci* is the c-p value for taxon *i* of soil nematodes and *pi* is the ratio of individuals to the total number of taxon *i* of free-living nematodes. MI evaluates functional changes in soil ecosystems after disturbance and restoration. A low MI indicates that the soil is highly disturbed, and conversely, a high MI indicates that the soil is less disturbed, there is a high proportion of K-strategists, and the nematode community is in a relatively stable state (Li et al., 2021; Ren et al., 2020).

6. Plant parasite index:


PPI=∑​vi×fi

(Bongers, 1990)

Where *vi* is the c-p value of taxon *i* of plant parasites soil nematodes, and *fi* is the ratio of individuals to the total number of individuals of taxon *i* of plant parasites. PPI is a maturity index that is specifically designed for the study of plant parasites. A high PPI indicates that plant parasites have more opportunities to feed on plants (Niu et al., 2020; Zhu et al., 2017).

7. Wasilewska index:


WI=(Ba+Fu)/Pp

(Wasilewska, 1994; Yeates, 2003)

where Ba, Fu and Pp are the number of bacterivores, fungivores and plant-parasites in the soil nematode community, respectively. WI was used to analyze the mineralization pathways in the soil food web.

8. Nematode channel ratio:


NCR=Ba/(Ba+Fu)

(Yeates, 2003)

NCR was used to characterize the importance of Ba and Fu in the decomposition channel. When the NCR is greater than 0.5, it indicates that the decomposition of soil organic matter is mainly dependent on the bacterial pathway, and on the contrary, the fungal pathway is dominant (Liu et al., 2022).

9. Enrichment index:


EI=100×[e/(e+b)]

(Ferris et al., 2001)

10. Structure index:


SI=100×[s/(s+b)]

(Ferris et al., 2001)

Where *e*, *b* and *s* denote the enriched, basal, and structural components of the food web, respectively. EI reflects the input of external nutrients and is used to assess the response of the food web to the available resources. SI denotes the connectivity, structural complexity, and length of the food chain of the soil food web. The larger the SI, the more complex the structure of the food web and the lesser resistance it has. EI and SI are considered simultaneously for assessing soil enrichment and food web development.

Nematode faunal analysis was performed based on EI and SI values of nematode communities at different elevations, and the results can provide a reference for soil food web status and soil environment (Chen et al., 2014).

### 2.6 Data analysis

A two-way ANOVA was used to examine the effects of elevation gradient, soil depth, and their interaction on soil physicochemical properties, soil nematode abundance, and ecological indices. A one-way ANOVA was used to test for differences in soil factors and nematode communities between different elevations and soil layers (LSD, α=0.05). Pearson correlation analysis and redundancy analysis (RDA) were used to analyze the relationships between soil nematode communities and soil physicochemical properties. Data organization and statistical analysis were performed in Microsoft Excel 2016 and IBM SPSS Statistics version 26.0 (SPSS Inc., Chicago, IL, USA), redundancy analysis (RDA) was conducted using Canoco 5.0 software, and plotting was done in GraphPad Prism 9 (GraphPad Software Inc., San Diego, CA, USA). All data were presented as mean ± standard error (SE).

## 3 Results

### 3.1 Soil physicochemical properties

Elevation and soil depth significantly affected soil physicochemical properties (**Table 1**). Elevation had a notable impact on BD, pH, SM, SOC, AK, TN, and AN across all soil layers. BD and pH both significantly decreased with increasing elevation, showing the highest values at E1, which were 0.97-1.03 g·cm−³ and 8.78-8.99, respectively. Conversely, SM, SOC, AK, TN, and AN exhibited a significant increasing trend with elevation. Notably, SOC and SM reached their highest values at E3 in the 10-20 and 20-40 cm soil layers, at 46.08 g·kg−¹ and 37.52%, respectively. AK, TN, and AN showed their highest values at E4, being 0.13-0.29 g·kg−¹, 2.37-4.35 g·kg−¹, and 0.09-0.16 g·kg−¹, respectively. AP, TC, and TP did not show significant changes with elevation in the 0-10, 20-40, and 10-20 cm layers. Soil properties significantly influenced by soil depth across the four elevations included pH, which increased significantly with depth and was highest in the 20-40 cm layer, with values ranging from 8.48 to 8.99. SOC and AK, on the other hand, significantly decreased with depth, showing the lowest values in the 20-40 cm layer, at 6.33-27.35 g·kg−¹ and 0.09-0.13 g·kg−¹, respectively. At the highest elevation (E4), SM significantly decreased with increasing soil depth, showing a 27.05% reduction. Furthermore, the interaction between elevation and soil depth significantly affected BD, SM, SOC, TN, and AN.

**Table 1:** Soil physical and chemical properties at different depths and elevations.

Depth (cm)	Elevation (m)	BD (g·cm^-3^)	SM (%)	pH	TC (g·kg^-1^)	SOC (g·kg^-1^)	TN (g·kg^-1^)	TP (g·kg^-1^)	AN (g·kg^-1^)	AK (g·kg^-1^)	AP (g·kg^-1^)
0-10	E1	0.97± 0.06Aa	31.51± 2.26Ac	8.78± 0.06Ba	0.44± 0.01Ac	11.06± 0.29Ad	1.30± 0.06Ad	0.44± 0.01Ac	0.04± 0.00Ad	0.20± 0.02Ab	0.01± 0.00Aa
	E2	0.88± 0.04Aa	36.03± 0.89Ac	8.52± 0.04Cb	0.47± 0.01Ab	21.38± 0.97Ac	2.33± 0.06Ac	0.47± 0.01Ab	0.08± 0.00Ac	0.20± 0.02Ab	0.01± 0.00Aa
	E3	0.62± 0.01Ab	48.50± 2.53Ab	8.58± 0.02Bb	0.48± 0.01Ab	43.81± 3.32Ab	2.95± 0.05Ab	0.48± 0.01Ab	0.11± 0.00Ab	0.23± 0.00Aab	0.02± 0.00Aa
	E4	0.42± 0.02Bc	65.14± 3.95Aa	8.13± 0.09Bc	0.54± 0.01Aa	61.82± 2.44Aa	4.35± 0.01Aa	0.54± 0.01Aa	0.16± 0.00Aa	0.29± 0.00Aa	0.02± 0.00Aa
10-20	E1	0.97± 0.03Aa	29.70± 1.88Ac	8.94± 0.02Aa	0.43± 0.01Aa	7.54± 0.41Bc	0.96± 0.06Bc	0.43± 0.01Aa	0.03± 0.00Bc	0.14± 0.00Bab	0.01± 0.00Ab
	E2	0.90± 0.05Aa	33.19± 2.27Ac	8.67± 0.02Bb	0.44± 0.01Ba	19.35± 1.25ABb	2.31± 0.07Ab	0.44± 0.01Ba	0.08± 0.00Ab	0.11± 0.02Bb	0.01± 0.00Ab
	E3	0.67± 0.04Ab	45.17± 3.17Ab	8.52± 0.05Bc	0.47± 0.05Aa	46.08± 2.09Aa	3.07± 0.19Aa	0.47± 0.05Aa	0.11± 0.01Aa	0.12± 0.00Bb	0.02± 0.00Aa
	E4	0.52± 0.02Bc	63.71± 2.32Aa	8.33± 0.02ABd	0.49± 0.02Ba	42.98± 3.27Ba	3.12± 0.31Ba	0.49± 0.02Ba	0.12± 0.01Ba	0.17± 0.00Ba	0.01± 0.00Bb
20-40	E1	1.03± 0.03Aa	27.80± 1.00Ac	8.99± 0.05Aa	0.42± 0.01Aab	6.33± 0.85Bc	0.81± 0.04Bb	0.42± 0.01Aab	0.03± 0.00Bb	0.09± 0.01Cb	0.01± 0.00Aa
	E2	0.83± 0.02Ab	36.15± 0.97Ab	8.82± 0.06Aab	0.45± 0.01Bab	16.66± 0.66Bb	2.09± 0.04Aa	0.45± 0.01Bab	0.07± 0.01Aa	0.09± 0.00Bb	0.03± 0.01Aa
	E3	0.61± 0.06Ac	47.52± 4.25Aa	8.75± 0.05Ab	0.40± 0.02Ab	20.74± 3.24Bab	1.66± 0.24Ba	0.40± 0.02Ab	0.07± 0.01Ba	0.10± 0.00Cb	0.01± 0.00Aa
	E4	0.77± 0.04Ab	42.72± 2.42Bab	8.48± 0.06Ac	0.45± 0.01Ba	27.35± 2.25Ca	2.37± 0.29Ba	0.45± 0.01Ba	0.09± 0.01Ba	0.13± 0.01Ca	0.01± 0.00Ba
Mixed-effect ANOVA	Elevations	85.58***	70.14***	71.76***	7.47**	186.35***	105.60***	7.47**	136.73***	23.25***	0.85ns
	Soil depths	5.43*	7.22**	27.72***	10.86**	67.92***	40.55***	10.86**	37.97***	159.68***	0.53ns
	Interactions	6.65***	6.16**	1.67ns	1.42ns	16.69***	8.92**	1.42ns	6.93**	2.21ns	0.41ns

E, Elevation; E1, 1750-1900 m; E2, 1900-2100 m; E3, 2100-2350 m; E4, 2350-2560 m. BD, Bulk density; SM, Soil moisture; TC, Total carbon; SOC, Soil organic carbon; TN, Total nitrogen; TP, Total phosphorus; AN, Available nitrogen; AP, Available phosphorus; AK, Available potassium. Data with different lowercase letters indicated significant differences among different elevations at the same soil layer (P< 0.05), while different uppercase letters indicated significant differences between different soil layers with the same elevation (P< 0.05). *P<0.05; **P<0.01; ***P<0.001; ns, not significant.

### 3.2 Composition and structure of soil nematode communities

**Figure 2:**
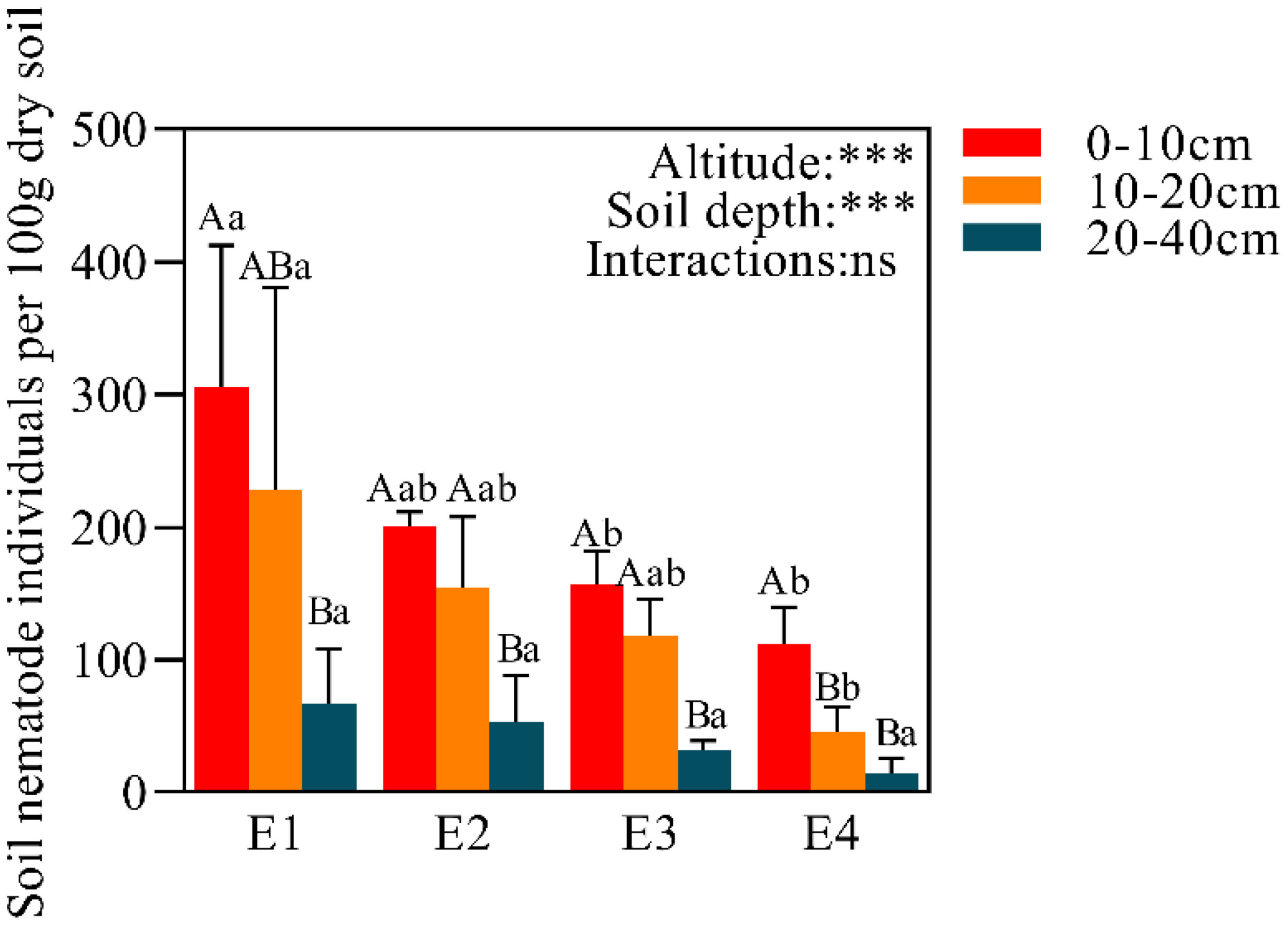
The total number of nematodes at different elevations in 0-10 cm,10-20 cm and 20-40 cm. Data with different lowercase letters indicated significant differences among different elevations at the same soil layer (P< 0.05), while different uppercase letters indicated significant differences between different soil layers with the same elevation (P< 0.05). ***P<0.001; ns, not significant.

A total of 1487 soil nematodes were identified on all elevation gradients (**Figure 2**), belonging to 27 families and 32 genera (**Table 2**). Both elevation and soil depth had significant effects on the total number of nematodes, but the interaction was not significant. The number of nematodes exhibited a decreasing trend along the elevation gradient for all soil layers, with the order E1 > E2 > E3 > E4. This trend was especially pronounced in the 0-10 cm and 10-20 cm soil layers, with decreases of 63.32% and 79.94%, respectively. Within each elevation, nematode abundance showed a decreasing pattern across soil depths, following the order 0-10 cm > 10-20 cm > 20-40 cm, with the 20-40 cm layer being significantly lower than the 0-10 cm layer, showing decreases of 78.06%, 73.59%, 79.79%, and 86.90%, respectively.

**Table 2:** Soil nematode community composition and c-p values of trophic groups at different elevations and depths.

Genus	c-p value	Dominance											
		E1			E2			E3			E4		
		D1	D2	D3	D1	D2	D3	D1	D2	D3	D1	D2	D3
Bacterivores,Ba													
*Monhystera*	1	++	++	++	++	++	+++	++	++	++	++	++	++
*Rhabditis*	1	++							+		++		
*Plectus*	2	++	++	++	++	++	++	++	++		++		
*Wilsonema*	2	+			+						++		
*Chiloplacus*	2	++	+++	++	++	++	+++	++	+++	++	++	+++	+++
*Acrobeles*	2										++	++	
*Cervidellus*	2	++	++	++	++	++	+++	+++	++	+++	++	++	++
*Teratocephalus*	3	++	++	+	++	+	+	+			++		
*Rhabdolaimus*	3	+			+	+	++	++	+		+	+	
*Prismatolaimus*	3	++	++	++	++	++	+++	++	++	++	++	++	++
Fungivores,Fu													
*Filenchus*	2								+		++		
*Aphelenchoides*	2	++	++	++	++	++	+++	++	+++	++	++	++	
*Aphelenchus*	2								++		++		++
*Tylencholaimus*	4									+++			
*Leptonchus*	4	++			+	+	++	++	+				
Omnivores-predators,Om													
*Tripyla*	3	++	++	++	++	++	+++	++	++	++	++		
*Mylonchulus*	4	++	++	+++	++	+++	++	++	++	++	++		
*Dorylaimus*	4	++	++	++	++	++	++	++	++		++		
*Eudorylaimus*	4	++	++	++	++	++	++	++	++	++	++	++	
*Thornia*	4	++	++	++	++								
*Pungentus*	5	+									++		
*Mesodorylaimus*	5		+		+	+					++	+	
*Longidorus*	5	+									++	+	
Plant-parasites,Pp													
*Ditylenchus*	2	++	++	++	+++	++	++	++	+++	++	++	++	
*Cephalenchus*	2	++		++				++	++				
*Helicotylenchus*	3	++	+++	++	++	+++	+++	+++	+++	++	++	+++	+++
*Amplimerlinius*	3	++	+	++	+			++	++		++		++
*Tylenchorhynchus*	3	++	++	++	++	++	++	++	++	++	++		
*Pratylenchus*	3	++		+	++						++		++
*Criconema*	3										++	+	++
*Trichodorus*	4	++	+					+	+	++			
*Axonchium*	5	++	++	++	+				++	++	++	++	

+++, The group with more than 10% of the total number of individuals; ++, The group with individual number of 1%—10%; +, The group with less than 1% of the total number of individuals. D, soil depth; D1, 0-10 cm; D2, 10-20 cm, D3, 20-40 cm.

Across the elevational gradient, the dominant taxon was *Helicotylenchus*, which accounted for 10.43% of the total number of nematodes; 17 genera such as *Mylonchulus* and *Dorylaimus* were common genera, which together accounted for 82.88% of the total number of nematodes; and 14 genera such as *Mesodoryrylaimus* and *Rhabditis* were rare genera, which together accounted for 6.69% of the total number of nematodes (**Table 2**). There were some differences in the dominant genera of soil nematode communities at different elevation gradients in each soil layer. In the 0–10 cm soil layer, there were no dominant genera in E1 and E4, mainly common genera, which accounted for 96.30% and 99.40% of the total number of soil nematodes, respectively; the dominant genera in E2 and E3 were *Ditylenchus*, *Cervidellus*, and *Helicotylenchus*, which accounted for 10.16%, 10.42% and 12.12%. In the 10-20 cm soil layer, the dominant genera in E1 were *Chiloplacus* and *Helicotylenchus*, which accounted for 10.25% and 11.13% of the total number of soil nematodes, respectively, while the dominant genera in E2 were *Mylonchulus* and *Helicotylenchus*, which accounted for 13.15% and 13.79% of the total number of soil nematodes, respectively; *Chiloplacus*, *Aphelenchoides*, *Ditylenchus*, and *Helicotylenchus* were the dominant taxa in E3, accounting for 11.35%, 10.21%, 13.05% and 11.91%, respectively; *Chiloplacus* and *Helicotylenchus* were the dominant taxa in E4, which accounted for 16.76% and 15.30%, respectively. In the 20–40 cm soil layer, *Mylonchulus* was the dominant genus in E1, accounting for 12.91% of the total number of soil nematodes; the dominant genera in E2 were *Monhystera*, *Chiloplacus*, *Cervidellus*, *Prismatolaimus*, *Aphelenchoides*, *Tripyla*, and *Helicotylenchus* with 18.24%, 26.42%, 30.82%, 10.06%, 26.42%, 13.84% and 10.06%, respectively; *Cervidellus* and *Tylencholaimus* were the dominant taxa in E3 with 12.63% and 10.55%, respectively; *Chiloplacus* and *Helicotylenchus* were the dominant taxa in E4 with 20.45% and 36.36%, respectively.

**Figure 3:**
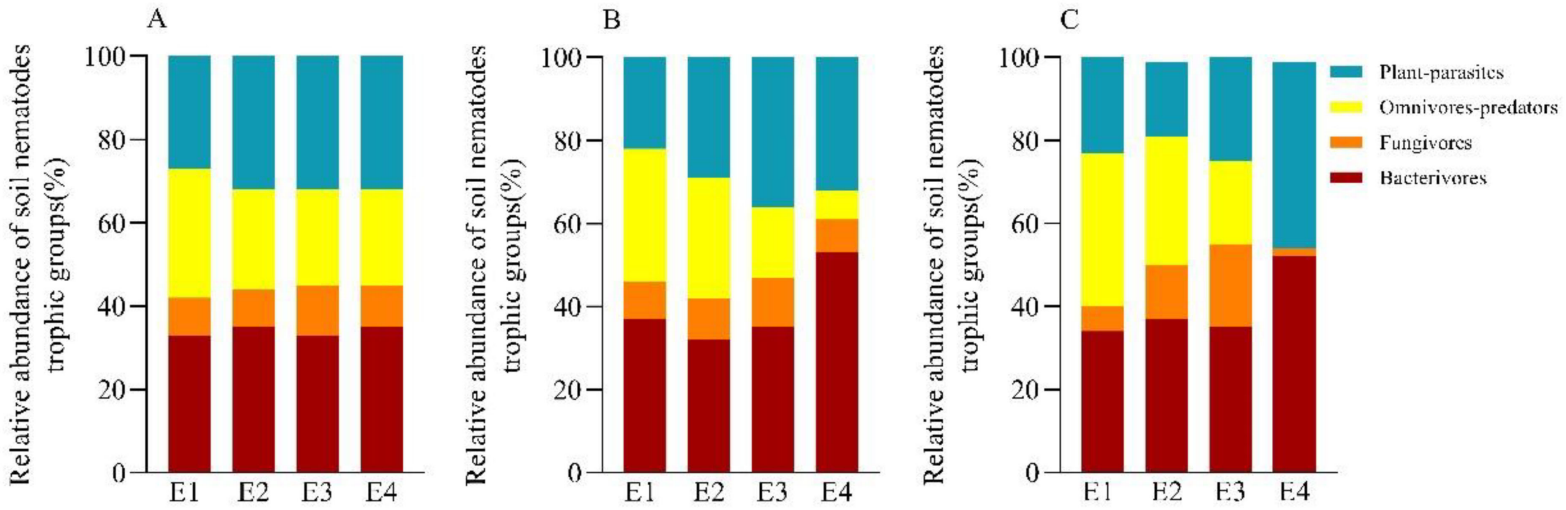
The relative abundance of soil nematode trophic groups at different elevations in 0-10 cm **(A)**, 10-20 cm **(B)**, and 20-40 cm **(C)**.

Among the different trophic taxa in each soil layer, bacterivore nematodes were the major trophic taxa with the highest total abundance at all elevations, accounting for 32.39% to 52.55% of the relative abundance at each elevation; followed by plant-parasites and omnivores-predators, which accounted for 18.24% to 45.45% and 7.29% to 37.25% of the relative abundance at each elevation; and fungivore nematodes were the lowest, accounting for only 2.27% to 20.02% (**Figures 3A–C**). Overall, the relative abundance of bacterivore nematodes and plant parasites increased with elevation, showing an increasing trend; the relative abundance of omnivores-predators was the opposite of that of bacterivore nematodes, decreasing with elevation; and the relative abundance of fungivore nematodes was in a “humpback pattern”, with a low-high-low pattern, with a maximum at E3.

### 3.3 Ecological indices and faunal analysis of soil nematode communities

**Figure 4:**
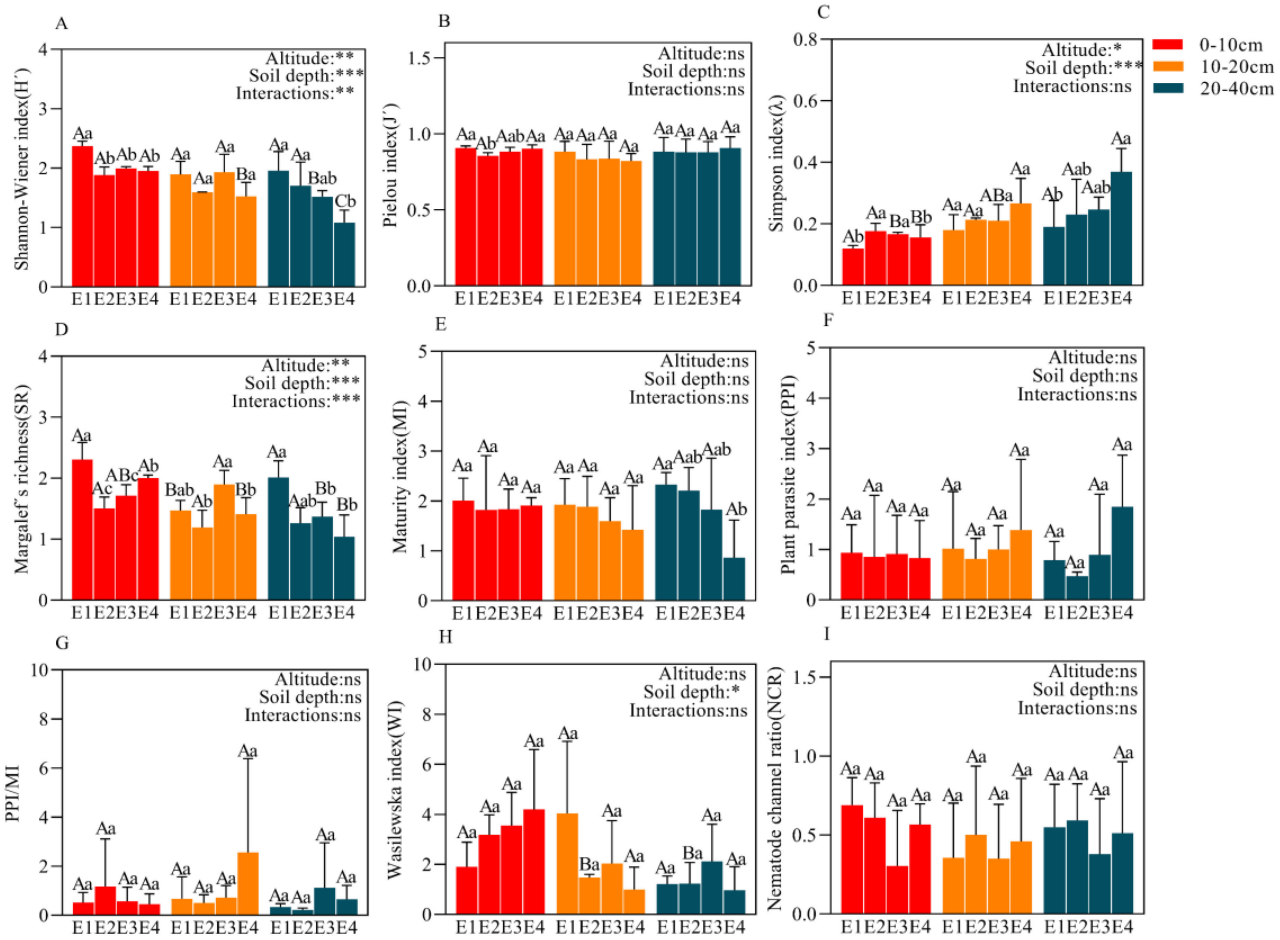
Ecological indices (H´, J´, λ, SR, MI, PPI, PPI/MI, WI, NCR) **(A–I)** of the soil nematode community at different elevations in 0-10 cm,10-20 cm, and 20-40 cm. Data with different lowercase letters indicated significant differences among different elevations at the same soil layer (P< 0.05), while different uppercase letters indicated significant differences between different soil layers with the same elevation (P< 0.05). *P<0.05; **P<0.01; ***P<0.001; ns, not significant.

Elevation and soil depth significantly affected H’, λ and SR, and their interaction also significantly influenced H’ and SR (**Figures 4A–D**). Within each soil layer, the patterns of H and SR changes with elevation were consistent, while λ showed the opposite trend. In the 0-10 and 20-40 cm soil layers, H and SR decreased with increasing elevation, whereas λ increased with elevation; there was no clear pattern in the 10-20 cm soil layer. Generally, across all elevations, H and SR decreased with soil depth, while λ increased.

In each soil layer, NCR was less than 0.5 at higher elevations (E3, E4) and greater than 0.5 at lower elevations (E1, E2); MI decreased with increasing elevation, being highest at E1; PPI and PPI/MI generally increased with elevation; WI increased with elevation in the 0-10 cm soil layer, showed the opposite pattern in the 10-20 cm soil layer, and followed a unimodal pattern in the 20-40 cm soil layer (**Figures 4E–I**). Across all elevations, MI increased with soil depth at E1, E2 and E3, but decreased at E4; PPI showed the opposite trend to MI with soil depth; WI was significantly higher in the 0-10 cm soil layer compared to the 10-20 and 20-40 cm layers (**Figures 4E, F, H**).

**Figure 5:**
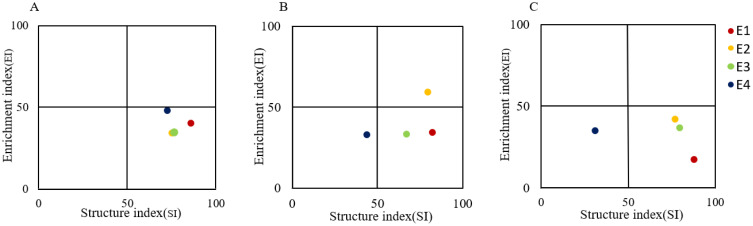
Nematode faunal analysis at different elevations in 0-10 cm **(A)** 10-20 cm **(B)** and 20-40 cm **(C)**.

Nematode fauna analysis showed that in the 0-10 cm soil layer, the soil nematode communities at all four elevation gradients were located in quadrant C. In the 10-20 cm soil layer, the nematode communities at E1 and E3 were in quadrant C, while those at E2 and E4 were in quadrants B and D, respectively. In the 20-40 cm soil layer, the nematode communities at E1, E2, and E3 were in quadrant C, whereas the community at E4 was in quadrant D (**Figures 5A–C**).

### 3.4 Relationship between soil nematode communities and soil properties

**Figure 6:**
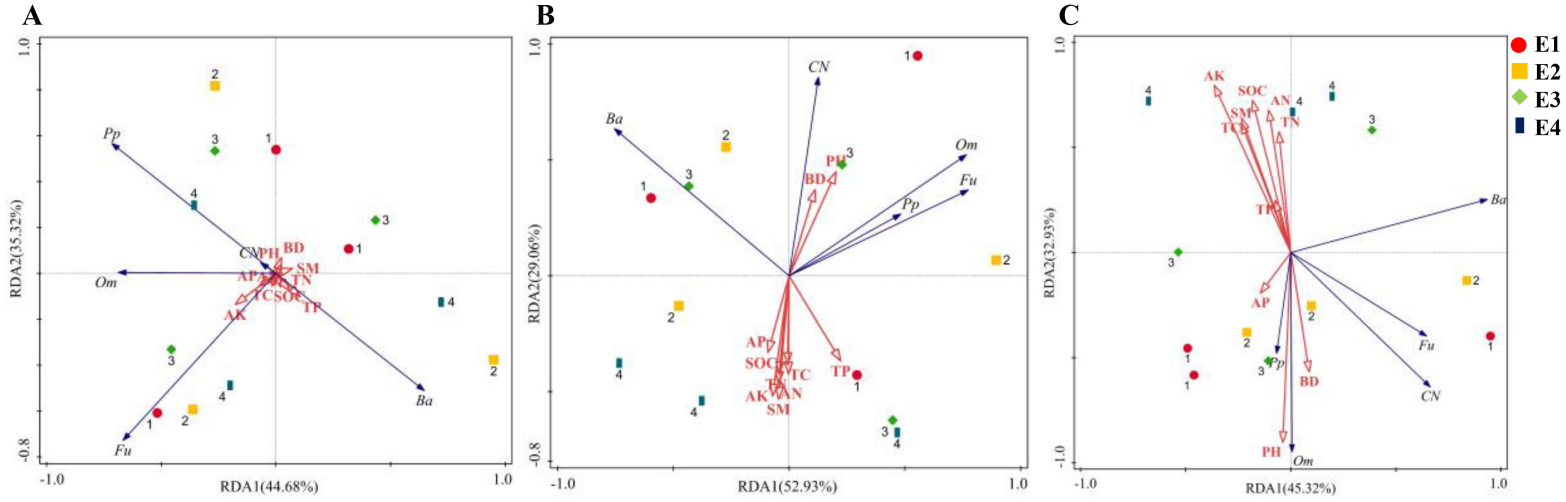
Redundancy analysis of soil nematode community with soil properties in 0-10 cm **(A)**, 10-20 cm **(B)**, and 20-40 cm **(C)**. BD, Bulk density; SM, Soil moisture; TC, Total carbon; SOC, Soil organic carbon; TN, Total nitrogen; TP, Total phosphorus; AN, Available nitrogen; AP, Available phosphorus; AK, Available potassium. Ba, Bacterivores; Fu, Fungivores; Om, Omnivores-predators; Pp, Plant-parasites. CN represents the sum of the number of individuals in the nematode community.

**Figure 7:**
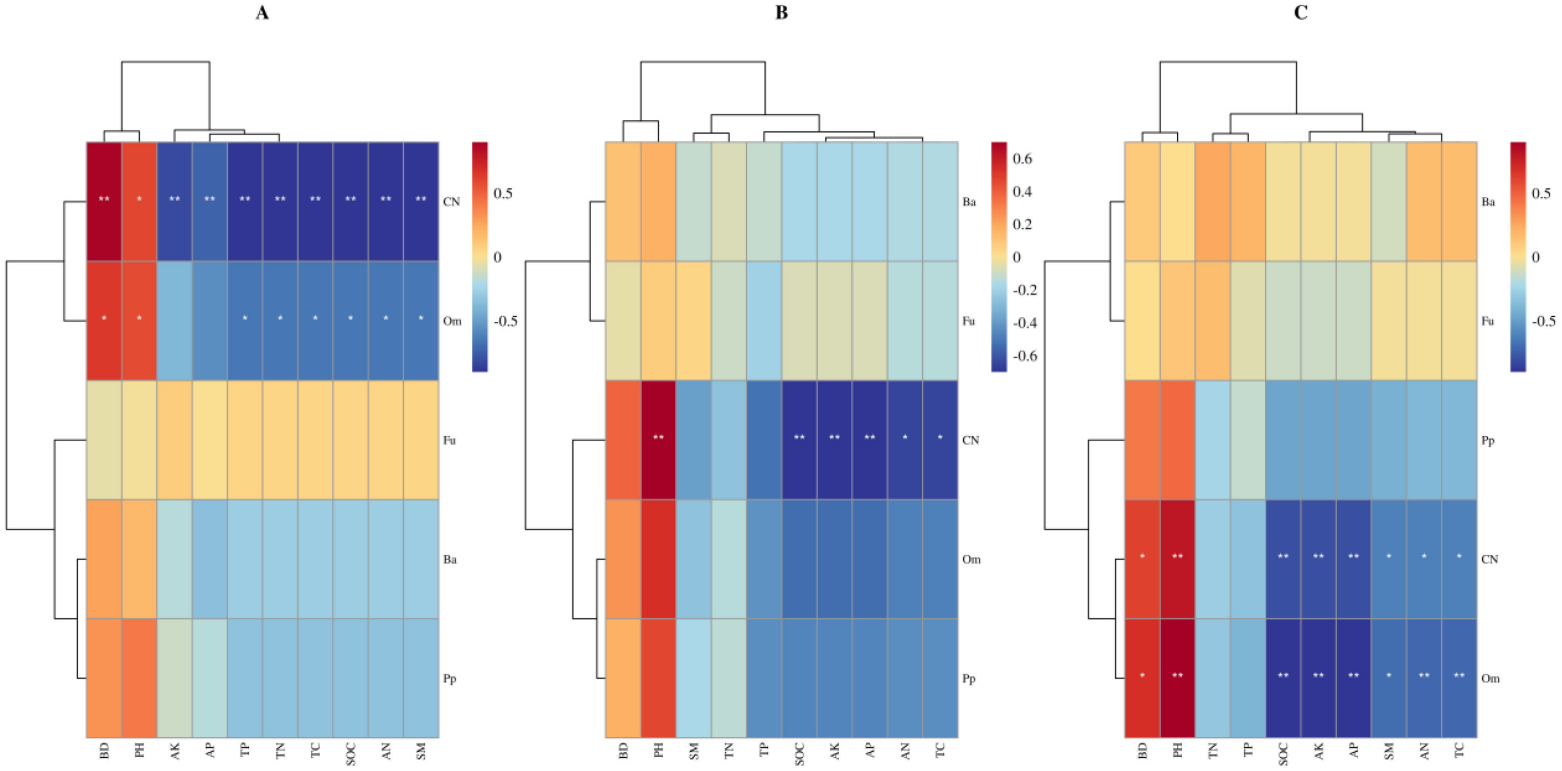
Correlation between different trophic groups of soil nematodes and soil properties in 0-10 cm **(A)**, 10-20 cm **(B)** and 20-40 cm **(C)**.

The total explanatory rates of variance for the RDA1 and RDA2 axes were 80.00%, 81.99% and 78.25%, respectively (**Figures 6A–C**). In the 0-10 cm soil layer, SM and pH were the key factors influencing soil nematode variation, with contribution rates of 14.0% and 13.0%, respectively. In the 10-20 cm soil layer, pH, SM, AP and SOC were the key factors, with contribution rates of 29.6%, 13.2%, 12.1% and 10.3%, respectively. In the 20-40 cm soil layer, pH, AK, SOC, AN, SM and BD were the key factors, with contribution rates of 28.5%, 28.0%, 22.1%, 19.4%, 15.9% and 12.6%, respectively. In the 0-10 cm soil layer, the total number of nematodes and omnivores-predators were significantly positively correlated with pH and BD, and significantly negatively correlated with SM (**Figure 7A**). In the 10-20 cm soil layer, the total number of nematodes was significantly positively correlated with pH, and significantly negatively correlated with SOC, AN, AK, AP and TC (**Figure 7B**). In the 20-40 cm soil layer, omnivores-predators were significantly positively correlated with pH and BD, and significantly negatively correlated with SOC, SM, AK, AP, AN and TC (**Figure 7C**).

## 4 Discussion

We observed a general decreasing trend in soil nematode numbers, diversity (H´) and richness (SR) along the elevational gradient and soil depth in Luoshan, Ningxia. This result is consistent with other studies such as Oakley in the northern of the Greater Khingan Mountains, China (Shen et al., 2023) and the Pir-Panjal Mountains of the Himalayas (Kashyap et al., 2022; Afzal et al., 2021; Choudhary et al., 2023), but different from the results from the Mount Ararat in Turkey, the number of soil nematode species was found to reach its maximum at medium elevation, showing a “single peak pattern” (Taylan et al., 2021). This may be attributable to the changes in temperature, SM, and elevations. Firstly, nematodes are very sensitive to changes in temperature (Afzal et al., 2021; Albright et al., 2020). Litter decomposition and nutrient release are key ecological processes linking the terrestrial and atmospheric carbon cycles, with a global average litter leaf carbon release of 0.69 yr^-1^ (Chen et al., 2024). As the elevational gradient increase and soil layer deepen, the temperature decreases, which reduces the rate of litter decomposition and microbial activity, leading to accumulation of soil organic matter and a decrease in nutrient levels, inhibiting nematode reproduction (Choudhary et al., 2023). Secondly, nematodes are very sensitive to changes in SM (Franco et al., 2021; Kardol et al., 2011). With higher elevation, increased precipitation will alter the coupling between aboveground and belowground, affecting nematode community diversity. In addition, the increase in soil water content may lead to hypoxia, reducing both nematode population and diversity (Afzal et al., 2021; Liu et al., 2019). Meanwhile, as the soil layer deepens and SM decreases, the activities of soil nematodes, especially free-living nematodes that are free in the water film, are limited, leading to a decline in nematode numbers and diversity at higher elevations (Tong et al., 2010). Thirdly, higher elevations present relatively hostile environments with relatively less resilient and sustainable ecosystems compared to lower elevations (Kashyap et al., 2022).

Interestingly, this study revealed that *Helicotylenchus*, with a cp value of 3, was the dominant taxon across the elevational gradient. However, differences across the dominant genera of soil nematode communities were observed in different elevational gradients within each soil layer, with a relatively high number of dominant nematodes with a cp value of 2 at higher elevations (Yeates and Bongers, 1999). This indicates that nematodes in Luoshan, Ningxia, predominantly consist of k-strategists, which have low reproduction rates and high sensitivity to disturbance (Wagner et al., 2015; Bongers, 1990). Conversely, at higher elevations, r-strategists among nematodes were relatively dominated, characterized by opportunism, high reproductive ability, and resistance to disturbance, which further indicates that higher elevations present more harsh environments and more susceptible to environmental disturbances compared to lower elevations (Tong et al., 2016). Additionally, the above-ground vegetation and the below-ground microenvironment vary greatly with elevation, and different environmental conditions and resource heterogeneity shape the distributional differences of the soil nematode community (Bardgett and van der Putten, 2014; Choudhary et al., 2023).

We found that among the various trophic taxa in each soil layer, bacterivore nematodes were the dominant trophic taxa, and their relative abundance with plant-parasites, increased with elevation, which is consistent with the findings of Choudhary et al. (2023). A plausible explanation is that, at high elevations, the dominance of forests with well-developed and dense underground root systems supports the survival of plant-parasites. Furthermore, among the bacterivore nematodes, the majority had a CP value of 2, indicating strong adaptation to the harsh environments. In addition, the relative abundance of omnivores-predators exhibited an inverse pattern to that of bacterivore nematodes, decreasing with increasing elevation, which is in line with Kashyap et al. (2022). This may be because omnivores-predators occupy a high trophic level in the soil food web and can switch to different prey as needed (Afzal et al., 2021). The relative abundance of fungivore nematodes showed a low-high-low “humpback pattern”, peaking at E3. This suggests that the elevational distribution patterns of soil nematode trophic groups vary and warrant further investigated. The dominance of bacterivore nematodes among all trophic groups across the elevational gradient suggests that the relatively fast decomposition model of fungal energy pathways prevails in Luoshan, Ningxia.

In this study, several key nematode species diversity indices and functional indices of nematode community structure were used to analyze soil nematode community structure. It was shown that the diversity index (H´), richness index (SR) and maturity index (MI) tended to decrease with elevation, while PPI/MI and PPI generally tended to increase with elevation gradient. This is consistent with previous studies (e.g., Choudhary et al., 2023), but differs somewhat from others (e.g., Afzal et al., 2021). The decrease in MI and the increase in PPI/MI indicate that soil ecosystems are subjected to increased disturbance in high elevation regions, reducing soil food web stability and the proportion of k- strategists nematodes (Chen et al., 2014; Ciobanu and Popovici, 2017). The overall increasing trend of PPI with elevation gradient indicates that plant-parasites had more plants to feed on and increased in number, corroborating the observations that the relative abundance of plant-parasites increased with elevation. Our study also found that the value of NCR decreased from greater than 0.5 to less than 0.5 with elevation, indicating a shift in the decomposition pathway of soil organic matter from the bacterial channel to the fungal channel (Liu et al., 2022). This shift occurs because coniferous forests predominate at high elevations in the Luoshan Mountain of Ningxia, where an abundance of C/N-containing compounds in the coniferous litter favors the fungal decomposition pathway. Similar to the study of Huo et al. (2021) and Afzal et al. (2021), this further supports the argument that bacterivore nematodes increased with elevation, while fungivore nematodes decreased at high elevation. The lower temperature and harsh environment at high elevations make it difficult and slow for some coniferous forests to decompose their withered leaves, enhancing the fungal-based energy flow in the ecosystem (Choudhary et al., 2023). In addition, our nematode faunal analysis revealed higher SI and lower EI values in E1, E2 and E3, while E4 had lower SI and lower EI values. This pattern indicates that soil was subjected to increased disturbance at higher elevations, the degree of structuring of the soil food web and nutrient enrichment was weakened, and the stability of the soil ecosystem declined, further supporting the above conclusions (Ciobanu and Popovici, 2017).

Elevation directly leads to the distribution of vegetation types, which modifies abiotic and biotic factors that, in turn, change the soil environment and soil physicochemical properties, ultimately affecting soil nematode communities (Bardgett and van der Putten, 2014; Fu et al., 2022; Kashyap et al., 2022). Our findings indicated that pH, BD, SM, SOC, AN, AP and AK were the main soil factors affecting soil nematode community composition. Pearson correlation analysis further corroborated the relationship between nematode community characteristics and soil physicochemical properties (**Figure 6**), well explained the differences in nematode communities at different elevations and soil depths. These results align with the findings of Shen et al. (2023) and Choudhary et al. (2023). It has also shown that soil pH and BD had significant effects on nematode communities, especially on omnivores-predators and nematode populations. Changes in soil pH may affect nematode physiology and metabolism, and may also indirectly affect nematode communities by altering the soil microenvironment (Li Q. M. et al., 2023). BD affects pore interstitial space, SM, and SOC, which have an impact on root growth and inter-root nutrient supply, and consequently affecting nematode populations. This impact is likely due to the simultaneous effect of BD and other variables, rather than a response to BD alone (Shen et al., 2023). SM was negatively correlated with nematode communities, probably because increased SM creates an anaerobic environment in the soil-water film, leading to hypoxia and a decline in nematode populations (Franco et al., 2021; Afzal et al., 2021). We also found that fast-acting nutrients such as AN and AP had a significant effect on nematodes. N and P are the major limiting factors in northern Chinese soils, and their nutrient-limited nature indirectly affects soil nematodes through plant roots and soil microbial activity (Shen et al., 2023). N enrichment has been shown to suppress nematode abundance and diversity (Liang et al., 2009), and ammonium has been shown to be toxic to a wide range of organisms. However, soil nematode trophic taxa respond differently to NH_4_^+^-N concentrations depending on feeding habits (Zhang et al., 2021).

## 5 Conclusion

Soil nematode distribution in Luoshan had a clear gradient effect. Our studied indicated that the number, diversity and richness of soil nematodes were significantly affected by elevation and soil depth, all exhibiting a decreasing trend with increasing elevation and soil depth. *Helicotylenchus* emerged as the dominant taxon, while bacterivore nematodes were the main trophic taxon, increasing with elevation. Key soil factors affecting soil nematode community composition included pH, BD, SM, SOC, AN, AP and AK. With increasing elevation, soil disturbance intensified, the degree of soil food web structuring and nutrient enrichment decreased, and the decomposition pathway shifted from bacterial to fungal. Our study provides strong results for the vertical distribution pattern of soil nematode communities in Luoshan, Ningxia, which can be used as the basic information for further study of soil biota in this mountainous area, and provides partial theoretical support for further exploration and study of the distribution pattern of soil nematode communities in the arid and semi-arid regions of Northwest China as well as globally.

## Data Availability

The original contributions presented in the study are included in the article/**Supplementary Material**. Further inquiries can be directed to the corresponding authors.
