# Supplementary material for: Distribution pattern of soil nematode communities along an elevational gradient in arid and semi-arid mountains of Northwest China
Source: Front Plant Sci. 2024 Oct 14;15:1466079. doi: 10.3389/fpls.2024.1466079 (PMC11523865; doi:10.3389/fpls.2024.1466079)
Supplement: Supplementary file 1 [file Table1.docx]

Table S1 Site characteristics of different elevations

| Elevation (m) | Longitude  (E) | Latitude  (N) | Vegetation type | Soil type | Dominant taxa |
| --- | --- | --- | --- | --- | --- |
| 1750-1900 | 106°15′21′′-106°15′45′′ | 37°15′11′′-37°15′19′′ | Perennial herbaceous steppe and semi-shrub steppe | Sierozem | *Stipa breviflora、Stipa grandis、Stipa tianschanica var. gobica、*  *Artemisia frigida、*  *Neotrinia splendens、*  *Oxytropis aciphylla、*  *Convolvulus tragacanthoides* |
| 1900-2100 | 106°16′25′′-106°16′39′′ | 37°15′42′′-37°15′49′′ | Evergreen coniferous, broad-leaved shrubs | Calcareous grayish-brown soil | *Juniperus sabina、Ostryopsis*  *davidiana、Spiraea lasiocarpa、Cotoneaster multiflorus、*  *Caragana korshinski、*  *Syringa oblata* |
| 2100-2350 | 106°16′57′′-106°16′58′′ | 37°16′3′′-37°16′39′′ | broadleaved deciduous forest | Gray Cinnamonic Soil, Sierozem | *Elaeagnus angustifolia、*  *Prunus sibirica、*  *Populus davidiana、*  *Betula platyphylla* |
| 2350-2560 | 106°16′52′′-106°16′53′′ | 37°17′7′′-37°18′6′′ | Cold temperate coniferous forest | Umbric Gray Cinnamonic Soil | *Picea crassifolia、*  *Pinus tabuliformis，*  *Picea crassifolia and Pinus*  *tabuliformis* |
